# *Botrytis cinerea* Transcriptome during the Infection Process of the Bryophyte *Physcomitrium patens* and Angiosperms

**DOI:** 10.3390/jof7010011

**Published:** 2020-12-28

**Authors:** Guillermo Reboledo, Astrid Agorio, Lucía Vignale, Ramón Alberto Batista-García, Inés Ponce De León

**Affiliations:** 1 Departamento de Biología Molecular, Instituto de Investigaciones Biológicas Clemente Estable, Montevideo 11600, Uruguay; greboledo@iibce.edu.uy (G.R.); aagorio@iibce.edu.uy (A.A.); lvignale@iibce.edu.uy (L.V.); 2 Centro de Investigación en Dinámica Celular, Instituto de Investigación en Ciencias Básicas y Aplicadas, Universidad Autónoma del Estado de Morelos, Cuernavaca 62209, Mexico; rabg@uaem.mx

**Keywords:** *Botrytis cinerea*, transcriptome, *P. patens*, angiosperms, ROS, cell wall degrading enzymes, transporters, virulence, secretome

## Abstract

*Botrytis cinerea* is a necrotrophic pathogen that causes grey mold in many plant species, including crops and model plants of angiosperms. *B. cinerea* also infects and colonizes the bryophyte *Physcomitrium patens* (previously *Physcomitrella patens*), which perceives the pathogen and activates defense mechanisms. However, these defenses are not sufficient to stop fungal invasion, leading finally to plant decay. To gain more insights into *B. cinerea* infection and virulence strategies displayed during moss colonization, we performed genome wide transcriptional profiling of *B. cinerea* during different infection stages. We show that, in total, 1015 *B. cinerea* genes were differentially expressed in moss tissues. Expression patterns of upregulated genes and gene ontology enrichment analysis revealed that infection of *P. patens* tissues by *B. cinerea* depends on reactive oxygen species generation and detoxification, transporter activities, plant cell wall degradation and modification, toxin production and probable plant defense evasion by effector proteins. Moreover, a comparison with available RNAseq data during angiosperm infection, including *Arabidopsis thaliana, Solanum lycopersicum and Lactuca sativa*, suggests that *B. cinerea* has virulence and infection functions used in all hosts, while others are more specific to *P. patens* or angiosperms.

## 1. Introduction

*Botrytis cinerea* is a necrotrophic fungus that causes grey mold and infects more than 1000 plant species, including model plants and many crops such as tomato, lettuce and berries [1,2,3]. It is found worldwide causing disease in fruits, flowers and leaves, leading to pre- and post-harvest crop losses [4,5,6]. *B. cinerea* is considered the second most important plant-pathogenic fungus, based on scientific and economic significance [2], and therefore it is an extensively studied plant pathogen. This fungus mainly enters the plant via direct penetration or through natural openings or wounds [7]. This ascomycete is a necrotrophic pathogen with a broad host range that kills the plant cells and colonizes the dead tissues to acquire nutrients [7]. *B. cinerea* stimulates reactive oxygen species (ROS) production by the plant and exploits the host programmed cell death (PCD) machinery to cause infection [8,9]. The infection strategies are well described and they rely on several virulence factors, including toxins and plant cell wall degrading enzymes (PCWDEs), necrosis-secreted proteins, transporter proteins and enzymes that protect the fungus from oxidative stress [10]. After landing on the plant surface, spores germinate and appresoria-mediated penetration involves the formation of a penetration peg needed to enter into the host cells [11]. CWDEs such as pectinases, including poly-galacturonases (PGs) and pectate and pectin lyases, as well as cellulases, xylanases, cutinases, lipases, and proteases, are produced to breach the plant surface, allowing plant tissue colonization and release of carbohydrates for consumption [9]. The high capacity for plant cell wall degradation and modification of glycoconjugates and polysaccharides by *B. cinerea* was demonstrated by the presence in its genome of 367 genes encoding putative CAZymes (Carbohydrate-Active enZymes) [12]. Most CWDEs and cutinases are encoded by multigenic families, and mutants in these genes do not always affect fungal virulence, indicating that some of these enzymes may have partly redundant functions [9,13,14,15,16]. Host cell death is facilitated by the action of fungal phytotoxins, including the two main toxins: botrydial and botcinic acid [17,18]. *B. cinerea* produces oxalic acid leading to acidification of the plant tissue [19], and mutants unable to produce this metabolite do not colonize the host tissues [20].

Plant cells recognize *B. cinerea* rapidly and mount a defense response activating the production of antimicrobial compounds and antimicrobial Pathogenesis-Related proteins (PRs), increasing the number of hormones such as salicylic acid (SA), jasmonic acid (JA), ethylene (ET) and brassinosteroids (BR), and other genes with different roles in defense [21]. In turn, *B. cinerea* suppresses plant defenses in early stages by producing small RNAs and effector proteins, which enables the fungus to establish inside the host tissues and accumulate biomass prior to the necrotrophic phase [22]. In addition to angiosperms, *B. cinerea* also infects and colonizes mosses in nature (*Polytrichum juniperinum*; [23]), and under axenic laboratory conditions (*Physcomitrium patens* (previously *Physcomitrella patens*); [24]). Mosses, liverworts and hornworts are bryophytes and, due to their key phylogenetic position as members of the sister lineage of vascular plants, they represent ideal organisms to perform evolutionary studies on defense mechanisms activated in plants in response to biotic stress. Fossil records suggest the existence of pathogenic fungi interacting with plants 400 million years ago [25]. These microorganisms might have imposed a selective pressure on early land plants, leading to the development of plant defense mechanisms to respond to microbial infection. Similar as in angiosperms, *B. cinerea* colonization leads to host cell death and necrosis of *P. patens* tissues [24,26]. After landing on the moss surface, spores germinate, germ tubes elongate and appressoria penetrate the plant cell cytoplasm directly through the plant leaf cell walls, or by invasion of the intercellular spaces between leaf cells leading to cytoplasm infection [27]. In response to infection, *P. patens* activates a defense response that includes the production of ROS, SA and the precursor of JA cis-oxophytodienoic acid (OPDA), reinforcement of the cell walls, and induction of genes encoding PRs and enzymes involved in secondary metabolism [24,26]. However, the activation of these defense mechanisms is not sufficient to stop *B. cinerea* growth and colonization, leading to plant decay. Recently, we have shown that *P. patens* responds to this fungal pathogen with transcriptional reprogramming of 3072 plant genes, which encode proteins involved in pathogen perception, signaling, transcription, hormonal signaling, secondary metabolic pathways and proteins with diverse role in defense against biotic stress [28]. To gain more insights into *B. cinerea* infection and virulence strategies displayed during moss colonization, we focused on the transcriptional response of *B. cinerea* genes during the infection process of *P. patens*. The results demonstrate that fungal genes encoding enzymes involved in ROS production and detoxification, CAZymes, and other virulence factors such as transporters, toxins and cell death inducing factors, were upregulated in *P. patens* tissues. Moreover, comparative analysis of *B. cinerea*-upregulated genes during *P. patens* and three angiosperms infections suggests that some genes involved in the infection process and virulence are commonly upregulated in all plant hosts, while other genes with these functions are more specific to *P. patens* or angiosperms.

## 2. Materials and Methods

### 2.1. B. cinerea, P. patens Inoculation and Microscopy

A *B. cinerea* strain isolated from lemon plants [24] was cultured on potato dextrose agar (PDA) at 22 °C, with a photoperiod of 16-h light/8-h dark. To inoculate *P. patens*, a spore suspension of *B. cinerea* was prepared from a 12-day-old culture. For this, *P. patens* Gransden wild type colonies were cultivated axenically on cellophane overlaid solid BCDAT medium (1.6 g L^−1^ Hoagland’s, 1 mM MgSO_4_, 1.8 mM KH_2_PO_4_ pH 6.5, 10 mM KNO_3_, 45 μM FeSO_4_, 1 mM CaCl_2_, 5 mM ammonium tartrate, and 10 g L^−1^ agar) as described by Ashton and Cove [29]. Moss colonies were grown at 22 °C under standard long-day conditions (16-h light/8-h dark regime under 60–80 μmol m^2^ s^−1^ white light). After 3 weeks of growth, colonies were sprayed with a 2 × 10^5^ spores/mL suspension of *B. cinerea* and each colony received approximately 5000 spores. Tissues were inoculated 5 h after the start of photoperiod. Three time points were analyzed; 4 h post inoculation (hpi), 8 hpi and 24 hpi. *B. cinerea* mycelium grown on PDA plates for 12 days was used as a control. Three independent biological replicates, consisting of 3 plates with 16 moss colonies each, at each infection time point, and mycelium grown on PDA plates were harvested for RNA extraction, immediately frozen in liquid nitrogen, and stored at −80˚C.

*B. cinerea* tissues were stained with 0.1% solophenyl flavine 7GFE 500 according to [26]. Fluorescence microscopy was performed with an Olympus BX61 microscope (Shinjuku-ku, Japan). Photographs were taken at 4 hpi, 8 hpi and 24 hpi.

### 2.2. RNA Extraction, RNA Sequencing, Data Processing and qPCR Analysis of P. patens-Infected Tissues

RNA was extracted using the RNeasy Plant Mini Kit, including a RNase-Free DNase I digestion in column (Qiagen, Germany). RNA quality control, library preparation, and sequencing were performed at Macrogen Inc. (Seoul, Korea). RNA integrity was checked before library preparation using an Agilent Technologies 2100 Bioanalyzer (Agilent Technologies). Libraries for each biological replicate were prepared for paired-end sequencing by TruSeq Stranded Total RNA LT Sample Prep Kit (Plant) with 1 μg input RNA, following the TruSeq Stranded Total RNA Sample Prep Guide, Part # 15,031,048 Rev. E. Sequencing was performed on Illumina platform (Illumina, CA, USA) by Macrogen Inc. (Seoul, Korea) to generate paired-end 101 bp reads, obtaining 41 to 64 M reads per sample with Q20 > 98.43%. RNA-seq processing steps were done through Galaxy platform (https://usegalaxy.org/). Raw reads quality was revised by FastQC software ver. 0.11.2 (http://www.bioinformatics.babraham.ac.uk/projects/fastqc/) and then preprocessed for both quality and adapter trimmings using Trimmomatic Version 0.38.0 software [30]. Additionally, to the default options, the following parameters were adjusted: adapter sequence TruSeq3 (paired-ended, for MiSeq and HiSeq), always keep both reads of PE, and SLIDINGWINDOW: 4:15 HEADCROP:12 MINLEN:50. Trimmed reads were mapped to the reference genome of *B. cinerea* isolate B05.10 (ASM14353v4) (http://fungi.ensembl.org/Botrytis_cinerea/Info/Index) using Hisat2 software [31]. The BAM files were obtained with Samtools View software ver. 1.9 and then sorted by name with Samtools Sort software ver. 2.0.3 [32], for further analysis. All raw RNA-Seq read data are deposited in the National Center for Biotechnology Information (NCBI) Short Read Archive (http://www.ncbi.nlm.nih.gov/sra/) under the BioProject accession code PRJNA647932.

Reads were counted using FeatureCounts software ver. 1.6.4 [33]. Additionally to default options, parameters were adjusted for: count fragments instead of reads, allow read to map to multiple features, and use reference sequence file GCA_000143535.4_ASM14353v4_genomic. Cluster analysis of replicates from each time point and control samples was performed by Principal Component Analysis (PCA) using pcaExplorer 2.16.0 software [34]. Differential expression analyses were performed using EdgeR software ver. 3.24.1 [35], with *p*-value adjusted threshold 0.05, *p*-value adjusted method of Benjamini and Hochberg [36] and Minimum log2 Fold Change 2. Counts were normalized to counts per million (cpm) with the TMM method and low expressed genes filtered for count values ≥ 3 in all samples. In this study, a false discovery rate (FDR) ≤ 0.05 was used to determine significant differentially expressed genes (DEGs) between *B. cinerea* grown on *P. patens* and *B. cinerea* grown on PDA (control), and expression values were represented by log2 ratio.

Expression level of 16 selected *B. cinerea* genes related to pathogenesis was analyzed to validate RNAseq results via quantitative reverse transcription PCR (RT-qPCR). cDNA was generated from 1 μg of RNA using RevertAid Reverse transcriptase (Thermo Scientific) and oligo (dT) according to the manufacturer’s protocol. RT-qPCR was performed using the QuantiNova Probe SYBR Green PCR Kit (Qiagen, Germany), according to manufacturer’s instructions, in an Applied Biosystems QuantStudio 3 thermocycler. Relative expression of each gene was normalized to the quantity of constitutively expressed BctubB, using the 2^−ΔΔCt^ method [37]. Gene expression of *B. cinerea* grown on plant was expressed relative to *B. cinerea* grown on PDA, with its expression level set to one. Each data point is the mean value of three biological replicates. The significance for quantitative gene expression analysis was determined with Student’s *t*-test using GraphPad Prism software ver. 8.0.2. *p*-values < 0.05 were considered statistically significant. Primer pairs used for qPCR analyses are provided in Supplementary Table S1. All primer combinations showed amplification efficiencies greater than 95%.

### 2.3. RNA-seq Data from B. cinerea Grown on A. thaliana, S. lycopersicum, and L. sativa and Comparison with B. cinerea Grown on P. patens

RNA-seq Illumina sequence reads from *B. cinerea* grown in *Arabidopsis thaliana* [38], *Lactuca sativa* (lettuce) [39], and *Solanum lycopersicum* (tomato) [40], were obtained from NCBI. These plant hosts were chosen since *A. thaliana* represents a model plant for angiosperms, and *S. lycopersicum* and *L. sativa* are two important crops affected by *B. cinerea*. In all cases data correspond to plant leaves inoculated with a spore suspension of *B. cinerea* strain B05.10, using 1 × 10^5^ spores/mL for *A. thaliana* (2000 spores per leaf), 2 × 10^5^ spores/mL for *S. lycopersicum* (6000 spores per leaf) and 5 × 10^5^ spores/mL for *L. sativa* (10.000 spores per leaf). The closest available time points after inoculation compared to our *B. cinerea* transcriptomes in *P. patens* were considered; *A. thaliana* 12 and 24 hpi, *S. lycopersicum* 16 and 23 hpi, and *L. sativa* 24 and 48 hpi. Hence, in this study the corresponding RNA-seq were analyzed: *A. thaliana* at 12 hpi (SRR3383472, SRR3383521, SRR3383545) and 24 hpi (SRR3383805, SRR3383815, SRR3383816), *L. sativa* at 24 hpi (SRA059059, split and extract data using barcode 24hpib1 CCGTCC, 24hpib2 GTCCGC and 24hpib3 GTGGCC) and 48 hpi (SRA059059, split and extract data using barcode: 48hpib1 CTTGTA, 48hpib2 TTAGGC and 48hpib3 GATCAG), *S. lycopersicum* at 16 hpi (SRR12676683, SRR12676682, SRR12676681) and 23 hpi (SRR12676681, SRR12676679, SRR12676678). RNA-seq processing steps were done through Galaxy platform (https://usegalaxy.org/) using the same pipeline as described before for *P. patens* RNAseq processing; setting TruSeq3 for single-ended in Trimmomatic 0.38.0 in accordance to Illumina single-end sequencing data available for *A. thaliana*, *L. sativa* and *S. lycopersicum*. Differential expression analyses were performed using EdgeR software ver. 3.24.1, using the same parameters as for *P. patens* analysis (*p*-value adjusted threshold 0.05, *p*-value adjusted by Benjamini and Hochberg method and Minimum log2 Fold Change 2). Counts were normalized to counts per million (cpm) with TMM method and low expressed genes filtered for count values ≥ 3 in all samples; a FDR ≤ 0.05 was used to determine significant differentially expressed genes (DEGs) between *B. cinerea* grown on *A. thaliana*, *L. sativa*, *S. lycopersicum* and *B. cinerea* grown on PDA (control), and expression values were represented by log2 ratio.

To compare DEGs from *P. patens* and the three studied angiosperms, hierarchical clustering analysis of expressed genes were performed on log2 Fold-Change expression values using the “hclust” tool from R package “stats” ver. 3.6.0. To visualize the obtained data, heatmap plots were performed using the “heatmap.2” tool from R package “gplots” ver. 3.1.0.

### 2.4. Functional Clasification and Comparison with B. cinerea Secretomes

To test for GO terms enrichment in different sets of DEGs, GO and functional annotations were assigned using G: profiler (http://biit.cs.ut.ee/gprofiler/); which use data retrieved from Ensembl database and fungi, plants or metazoa specific versions of Ensembl Genomes [41]. Only annotated genes were used and GO terms with a FDR ≤ 0.05 were considered for our analysis.

*B. cinerea* DEGs obtained in the different plant species (*P. patens* and angiosperms) were compared with previously published *B. cinerea* secretomes [42,43]. Candidate effectors were searched for among these genes using EffectorP ver 2.0 (http://effectorp.csiro.au/).

## 3. Results

### 3.1. B. cinerea Differentially Expressed Genes during P. patens Infection

*P. patens* consists of juvenile proto-nemal filaments that further develop into leafy gametophores with rhizoids [44]. Proto-nemal filaments and leaves were infected by *B. cinerea*, as can be visualized by solophenyl flavine staining (Figure S1). *B. cinerea* infection process starts with the germination of spores at the plant surface at 4 h post inoculation (hpi), and continues with germ tubes elongation and formation of terminal swollen structures called appressoria at 8 hpi. Finally, hyphae proliferate and protonemal and leaf cells are invaded at 24 hpi. Accordingly, *B. cinerea* starts to enhance biomass production at 8 hpi, reaching high levels at 24 hpi [26]. Symptoms development were visible at 24 hpi with typical browning and maceration of the tissue (Figure S1).

**Figure 1:**
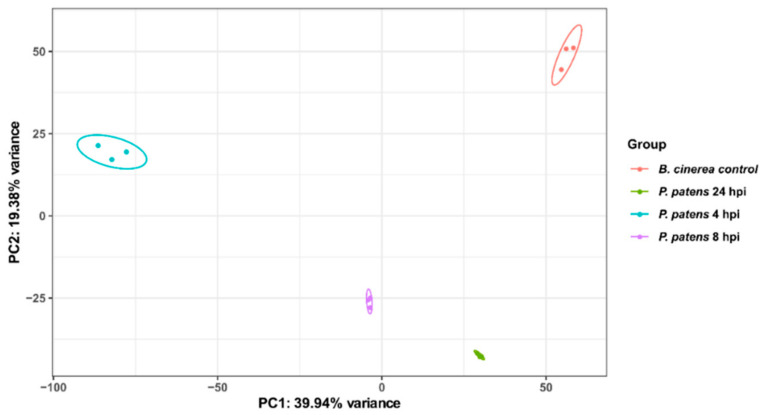
Analysis of *B. cinerea* RNA-Seq data during *P. patens* infection by principal component analysis (PCA). Variation among the three biological replicates per sample (*P. patens* 4 hpi, *P. patens* 8 hpi, *P. patens* 24 hpi and *B. cinerea* control), is shown. Colored dots denote each biological replicate.

In order to identify *B. cinerea* molecular players involved in virulence and *P. patens* colonization, we performed transcriptional profiling of these three stages. Samples of *B. cinerea* infecting *P. patens* tissues (4, 8 and 24 hpi) and *B. cinerea* grown on PDA medium (control) generated a total of 312,737,769 clean reads after removing adapter sequences and low-quality reads (Table S2). Considering all samples, 0.12% to 92.86% of the reads in the libraries mapped successfully to the genome of *B. cinerea* (nuclear and mitochondria). Reads mapped uniquely to *B. cinerea* nuclear genome were considered for further analyses; 98,929 at 4 hpi, 796,752 at 8 hpi, 4,261,033 at 24 hpi and 53,254,525 for control *B. cinerea* samples. Biological variability within replicates was analyzed by principal component analysis (PCA). As shown in Figure 1, the first principal component (PC1) accounted for 40% of the total variation and separates the three time points (4, 8 and 24 hpi), and the control *B. cinerea* samples. Variability between biological replicates was very low as indicated.

**Figure 2:**
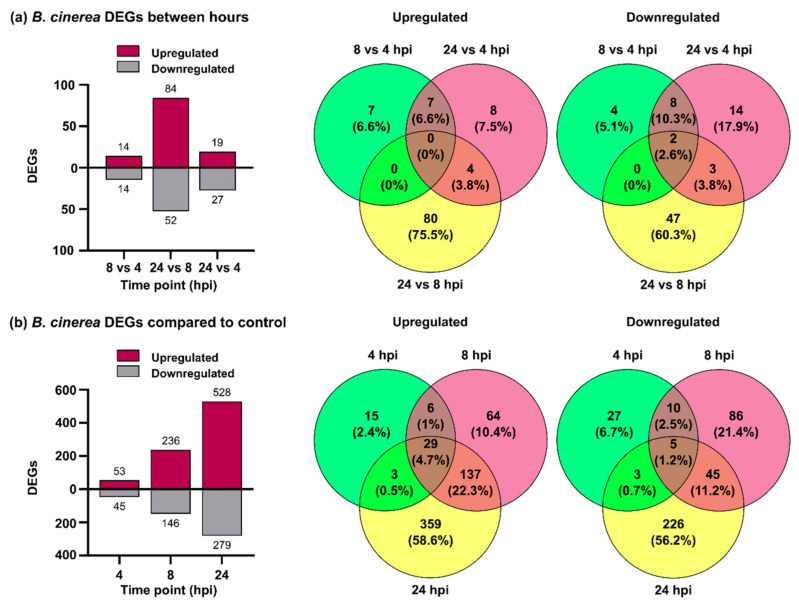
Differentially expressed *B. cinerea* genes during *P. patens* infection. (**a**) Number of differentially expressed genes (DEGs) and Venn diagrams from *B. cinerea* comparisons at 8 vs. 4 hpi, 24 vs. 8 hpi and 24 vs. 4 hpi. (**b**) Number of DEGs and Venn diagrams from *B. cinerea* at 4, 8 and 24 hpi compared with potato dextrose agar (PDA) samples. Log2 FC ≥ 2.0 or ≤ −2.0 and false discovery rate (FDR) ≤ 0.05 were considered for DEGs identification. In Venn diagrams, the overlap of expressed fungal genes can be observed.

Differential expression was first analyzed between *B. cinerea* genes expressed *in planta* during the different infection time points. In total, 184 *B. cinerea* genes were differentially expressed, 106 were upregulated and 78 were downregulated (Figure 2a; Table S3). In the 8 versus 4 hpi comparison, 14 differentially expressed genes (DEGs) were upregulated and 14 DEGs were downregulated. The number of DEGs increased when 24 hpi was compared with 8 hpi; 84 and 52 DEGs were upregulated and downregulated, respectively. Finally, the comparison of 24 versus 4 hpi showed 19 upregulated and 27 downregulated DEGs. Given the low number of *B. cinerea* DEGs, we compared each time point of infection with PDA grown mycelium in order to obtain more information on the infection process. In total, 1015 *B. cinerea* DEGs were obtained and among them 613 were upregulated and 402 downregulated (Figure 2b; Table S3).

Based on this finding, we decided to consider the 1015 DEGs for further analyses. *B. cinerea* DEGs increased over time and more upregulated genes than downregulated genes were observed at the three time points. The number of upregulated *B. cinerea* genes increased from 53 at 4 hpi, to 236 at 8 hpi, and finally to 528 at 24 hpi. Among downregulated genes, 45, 146 and 279 were identified at 4, 8 and 24 hpi, respectively. A high proportion of upregulated genes present at 4 hpi were also identified at 8 hpi and 24 hpi (71% and 78%), and similarly 70% of upregulated genes at 8 hpi were also upregulated at 24 hpi.

### 3.2. B. cinerea Genes Encoding Secreted Cell Wall Degrading Enzymes and Other Virulence Factors Are Induced during P. patens Infection

Gene ontology (GO) term enrichment analysis and manual inspection of the identified DEGs were performed to identify the biological processes (BP), molecular function (MF) and cellular compartment (CC) mostly affected in *B. cinerea* during moss infection (Figure S2; Table S4). Enriched downregulated processes include terms related to ribonucleotide and nucleotide binding at 4 hpi, transporter activities at 4 and 8 hpi, and ion binding activities at 8 and 24 hpi. In addition, downregulated genes were also enriched in GO terms such as tetrapyrrole and heme binding, oxidoreduction processes and oxidoreductase activities, and FAD binding and monooxygenase activity at 24 hpi. For upregulated genes, GO terms enrichment at 4 hpi were related to organic acid and oxoacid metabolic processes, amino acids metabolic and biosynthetic processes, oxidoreduction processes such as nicotinamide adenine dinucleotide (NAD) binding, oxidoreductase activities acting on different compounds, and transporter activity. At 8 hpi, the most significantly enriched GO terms for upregulated genes were those involved in degradation of various cell wall components, including hydrolase activities hydrolyzing O-glycosyl compounds or acting on glycosyl bonds, PG activity, catalytic activity and carbohydrate metabolic process. All these GO terms were also enriched for upregulated genes at 24 hpi, and other GO terms included carbohydrate binding, hydrolase activity, polysaccharide binding, cellulose binding, alpha-L-arabinofuranosidase activity, cutinase activity, pectate lyase activity, carbon-oxygen lyase activity acting on polysaccharides, and arabinose metabolic process. In addition, intrinsic components of membrane were present at all time points, while the extracellular region was a common GO term for upregulated genes at 8 and 24 hpi, which is consistent with secretion of hydrolytic enzymes to degrade the plant cell wall. These results show that GO enrichment analysis also provided insights into temporal characteristics of fungal pathogenesis.

**Figure 3:**
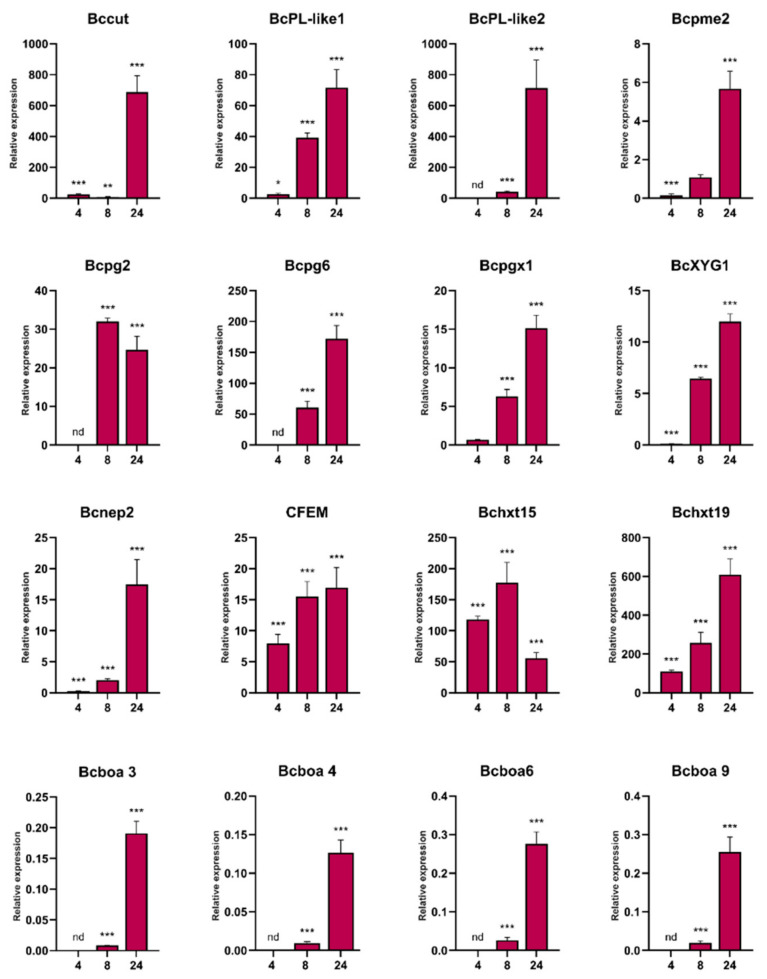
Validation of *B. cinerea* differentially expressed genes during *P. patens* infection by quantity reverse transcription RT-qPCR at 4, 8 and 24 hpi. The expression levels of *B. cinerea* genes infecting *P. patens* at the indicated time points are relative to the level of expression of *B. cinerea* grown in PDA. Genes are represented by the corresponding abbreviation as indicated in Table S1. *BctubB* was used as the reference gene. Results are reported as means ± standard deviation (SD) of three samples for each treatment. Asterisks indicate a statistically significant difference between *B. cinerea* grown in *P. patens* and in PDA (Students *t*-test, * *p* < 0.5, ** *p* <0.01; *** *p* < 0.005). Abbreviation: nd: no data.

We further focused on upregulated genes since they could encode proteins involved in *B. cinerea* pathogenesis. In total, 29 DEGs were commonly upregulated at all time points, including genes encoding several major facilitator superfamily (MFS) transporters, two sugar transporters (Bchxt19 and Bchxt15), a nitrate reductase (BcniaD), a nitrite reductase (BcniiA), several types of hydrogenases, a common fungal extracellular membrane (CFEM) domain-containing protein (Bcin09g02270), a chitinase, and several hypothetical proteins. In addition, genes involved in the catabolic pathway of D-galacturonic acid [45], which is the major component of pectin polysaccharides [46], were upregulated during the infection process, including those encoding a 2-keto-3-deoxy-L-galactonate aldolase (Bclga1), galactonate dehydratase (Bclgd1) and galacturonate reductase (Bcgar2). To validate the expression profiles of the RNA-Seq data, some of these genes and others related to pathogenesis were selected for analysis using RT-qPCR. The results obtained by the two techniques showed a strong correlation (R2 = 0.9882) (Figure 3; Table S5). Among the common 137 upregulated genes present at 8 and 24 hpi, virulence components such as cutinases and secreted proteins including CAZymes involved in pectin (mainly homogalacturonan), hemicellulose (mainly xyloglucan) and cellulose degradation were identified, as well as Bcnep1 and Bcnep2 (Necrosis- and Ethylene-inducing Proteins), glutathione peroxidase and glutathione S-transferase (GST) (Table S6). At 8 hpi upregulated genes encode PGs (Bcpg2, Bcpg4, Bcpg6, Bcpgx1 and a second exo-PG), pectate lyases, pectin lyases, beta-glucosidases, a xyloglucan endo-β-glucanase (BcXYG1), an endo-beta-1,4-glucanase, a xylanase, cellobiose dehydrogenases, a cello-bio-hydrolase, and arabino-furanosidases (Table S6). At 24 hpi, the number of upregulated genes encoding enzymes for homogalacturonan degradation increased to seven PGs (Bcpg2, Bcpg4, Bcpg5, Bcpg6, Bcpgx1 and two other exo-PGs), four pectate lyases, four pectin lyases and a pectin-esterase. Genes encoding enzymes that target xyloglucan were upregulated at 24 hpi, included β-glucoside gluco-hydrolase, three beta-glucosidases and BcXYG1 (Table S6). In addition, genes encoding enzymes for xylan degradation were also upregulated at 24 hpi, including Bcxyn11A, Bcxyn11C and three other xylanases. Genes encoding secreted enzymes acting on rhamnogalacturonans backbone, xylan backbone, mannans, cellulose, and side-chains were also upregulated at 24 hpi. These DEGs included glycoside hydrolases, rhamnogalacturonases, beta-glucosidase, mannosidases, glucanases and cello-bio-hydrolase, arabino-furanosidase and beta-galactosidases, among others. In addition, several peptidases were upregulated at 8 and 24 hpi. Taken together, these results support the important role played by these fungal enzymes during *P. patens* infection.

Surprisingly, genes encoding enzymes involved in botcinic acid synthesis were downregulated at 8 and 24 hpi, indicating higher expression levels in fungi growing on PDA medium. However, when DEGs expressed *in planta* were compared, several genes involved in botcinic acid production were upregulated at 24 hpi compared to 8 hpi, including Bcboa3, Bcboa4, Bcboa5, Bcboa6, Bcboa7 and Bcboa9 (Table S3; Figure 3). Other genes that increased *in planta* during the progression of infection (24 compared to 8 hpi), included those encoding cutinases, PGs, a pectin lyase, Bcnep2, and MSF transporters, among others (Table S3).

### 3.3. Genes Encoding B. cinerea Virulence Factors and Candidate Effectors Are Upregulated during Moss Infection

In addition to CWDEs genes, other genes encoding virulence factors and known effectors were present among the upregulated *B. cinerea* genes during *P. patens* infection. These included the cell death inducing factors Bcnep1 and Bcnep2, the necrosis-inducing xylanase Xyn11A and xyloglucanase BcXYG1, as well as the necrosis inducing Bcpg2. Several genes encoding CFEM domain-containing proteins were also upregulated during moss infection, including CFEM1, which is important for pathogenesis (Table S6). Genes encoding two secreted CalciNeurine-Dependant (CND1 and CND3) genes with putative virulence functions, peptidases and proteinases were also upregulated in moss tissues, including secreted aspartic proteinase (Bcap9), peptidases and a metalloproteinase (Deuterolysin; Bcmp1). We searched for effectors among the upregulated *B. cinerea* DEGs encoding secreted fungal proteins, using EffectorP (Table S6). Eight candidate secreted effectors were identified, including Pectin lyase (Bcin04g00470), BcNEP1, the small secreted BcSSP2A, two ribonucleases (Bcin07g03720 and Bcin05g04720), and three hypothetical proteins. One of these hypothetical protein-encoding genes, Bcin04g03920, showed very high expression levels at 8 and 24 hpi. These results indicate the involvement of virulence factors during *P. patens* infection, and suggest that candidate secreted effectors may interfere with plant defenses.

### 3.4. Comparison of B. cinerea DEGs during the Interaction with P. patens and Three Different Angiosperms

**Figure 4:**
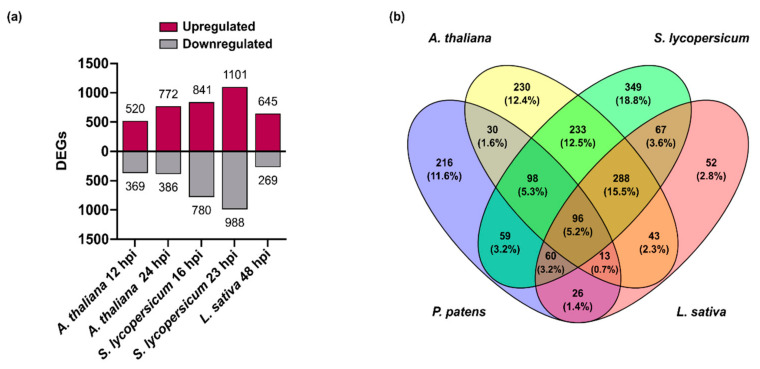
Differentially expressed *B. cinerea* genes during *P. patens* and angiosperms infection. (**a**) Number of differentially expressed genes (DEGs) from *B. cinerea* grown in different plant species at the indicated time points. (**b**) Venn diagram of *B. cinerea* upregulated DEGs in different plant species, showing overlap of upregulated fungal genes. *P. patens*: *B. cinerea* DEGs at 8 and 24 hpi; *A. thaliana*: *B. cinerea* DEGs at 12 and 24 hpi; *S. lycopersicum*: *B. cinerea* DEGs at 16 and 23 hpi; and *L. sativa*: *B. cinerea* DEGs at 48 hpi. For the Venn diagram, DEGs had log2 FC ≥ 2, FDR ≤ 0.05, and were expressed differentially in at least one time point for each plant species condition.

The expression pattern of *B. cinerea* genes encoding virulence factors and other proteins involved in the infection process of *P. patens* was compared with gene expression pattern during angiosperms infection, using available RNAseq data that were reanalyzed in this work. A summary of the number of reads mapped to the *B. cinerea* genome from each biological replicates in the different plant hosts is provided in Table S2. The percentage of reads that uniquely mapped to the *B. cinerea* genome relative to the total number of reads, in each sample, were in general similar between species, except for *P. patens* at 4–8 hpi and *L. sativa* at 24 hpi (lower percentage of reads), and *S. lycopersicum* at 23 hpi (higher percentage of reads). The number of *B. cinerea* genes targeted by these reads and the number of genes that passed FDR ≤ 0.05, for each plant species are shown in Table S7. Based on the low number of reads and DEGs in *L. sativa* 24 hpi, this time point was discarded for further analyses. PCA showed that PC1 accounted for 38% of the total variation and segregated *P. patens* from angiosperms (Figure S3). In addition, PC1 also allows the differentiation between early time point in angiosperms (*A. thaliana* 12 hpi and *S. lycopersicum* 16 hpi) and later time points (*A. thaliana* 24 hpi, *S. lycopersicum* 23 hpi and *L. sativa* 48 hpi). This result suggests that the transcriptome state of *B. cinerea* during early infection of the different angiosperms is comparable, and a similar scenario occurs within the late time points. The number of *B. cinerea* DEGs with |log2 FC| ≥ 2 in *A. thaliana* were 520 upregulated and 369 downregulated genes at 12 hpi, and 772 upregulated and 386 downregulated genes at 24 hpi. *S. lycopersicum* has 841 upregulated and 780 downregulated DEGs at 16 hpi and 1101 upregulated and 988 downregulated genes at 23 hpi, while *L. sativa* has 645 upregulated and 269 downregulated DEGs at 48 hpi (Figure 4a; Tables S8–S10).

**Figure 5:**
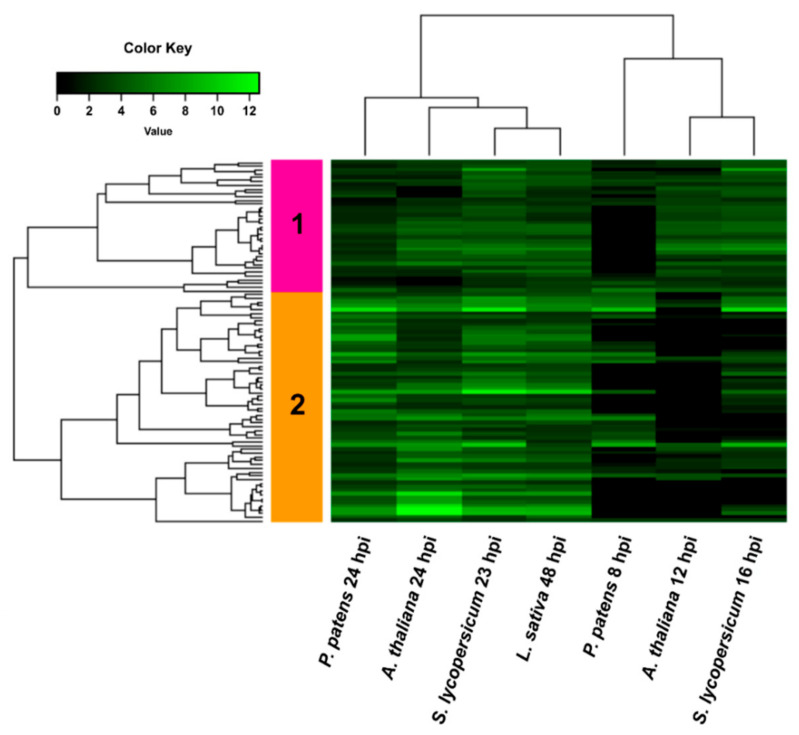
Heatmap of hierarchical clustering of common upregulated *B. cinerea* DEGs during infection of *P. patens* and different angiosperms. DEGs correspond to the 96 common upregulated genes of *B. cinerea* grown in all plant species. Selected DEGs had log2 FC ≥ 2, FDR ≤ 0.05, and were expressed differentially in at least one time point for each plant species. For samples where no significant difference with control was observed (FDR > 0.05) or no data was available, the log2 FC was assigned as cero. See Table S11 for complete information.

We first centered our analysis in the upregulated *B. cinerea* DEGs and show that 96 DEGs were commonly upregulated in all hosts, while 216 and 288 DEGs were only upregulated in *P. patens* and in the three angiosperms, respectively (Figure 4b). The 96 common upregulated DEGs showed an expression profile that separated samples into two groups by hierarchical clustering, one comprising the early time points and a second that included the late time points of infection in the different hosts (Figure 5; Table S11).

**Figure 6:**
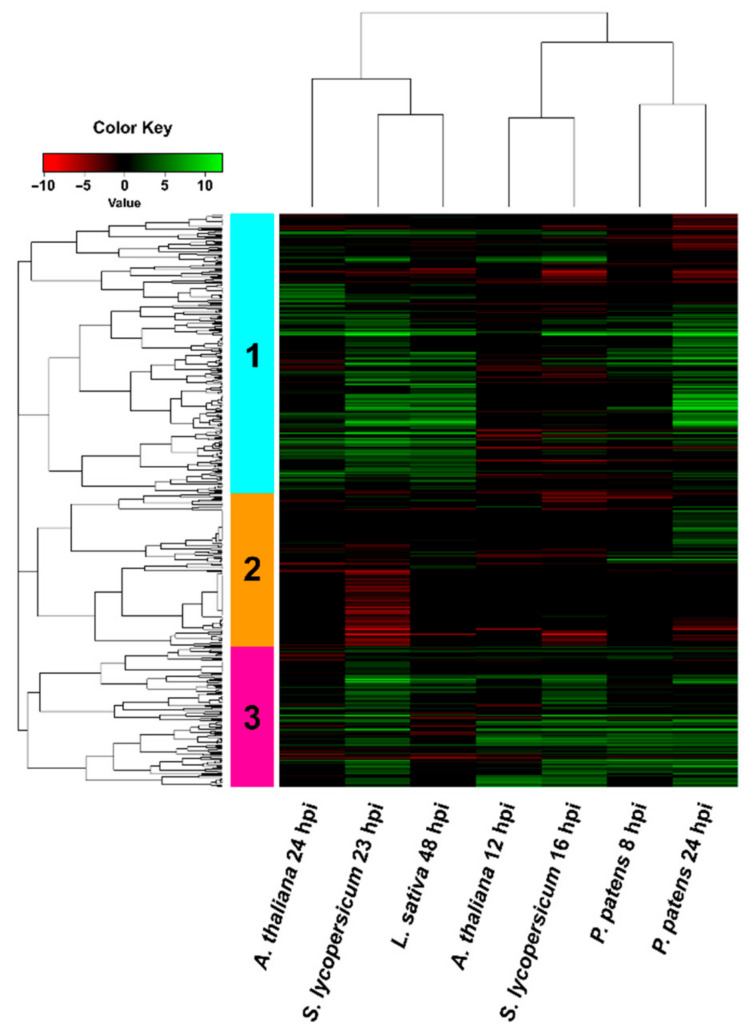
Hierarchical clustering (heat map) showing genes encoding secreted *B. cinerea* proteins during infection of *P. patens* and different angiosperms. Only DEGs that passed FDR ≤ 0.05 were considered in the analysis. For samples where no significant difference with control was observed (FDR > 0.05) or no data was available, the log2 FC was assigned as cero. See Table S12 for complete information.

In addition, two gene clusters with different expression patterns were identified; cluster 1 included DEGs that were equally upregulated at early and late time points, while cluster 2 showed a higher number of upregulated DEGs during the late time points of infection compared with early time points. Common upregulated *B. cinerea* genes in all plant species encoded proteins with oxidoreductase activity, virulence factors (NEP2 and CFEM containing protein), transferases, transporters and hypothetical proteins, distributed in both clusters. Cluster 2 included several genes encoding secreted CAZymes such as Bcpg2, Bcpg6, pectin lyases, pectate lyase, rhamnogalacturonan acetyl-esterase, α-L-rhamnosidase, α-galactosidase, β-galactosidase, α-N-arabino-furanosidase, and Bcxyn11A. Based on this result, we looked into more detail to the expression pattern of genes encoding predicted secreted proteins of *B. cinerea* during *P. patens* and angiosperm infection (Figure 6; Table S12). Hierarchical clustering grouped the expression pattern of secretome-encoding genes into a group that included the late time points of angiosperms, and a second group including both time points of *P. patens* and the early time points of angiosperms. In addition, clustering identified genes with three expression patterns, cluster 1 has the highest number of upregulated *B. cinerea* DEGs. We focused in this cluster and observed that *B. cinerea* DEGs were detected in some but not all hosts. Expression patterns of fungal DEGs were similar during infection of *S. lycopersicum* 23 hpi and *L. sativa* 48 hpi, while expression profiles of fungal genes in angiosperms and *P. patens* differ at late infection stages.

Cluster 1 also included DEGs that are commonly upregulated in all plant species such as the previously mentioned Bcpg2, Bcpg6, pectate lyase, pectin lyase and other CAZymes, which were among the highest upregulated DEGs in all plant species. In addition, several CAZymes genes were only upregulated at late time points in all plant species except *A. thaliana*, including genes encoding endoglucanases, endo-β-mannosidase, rhamnogalacturonase, α-L-arabino-furanosidase, a cellobiohydrolase, a glycoside hydrolase and a xylanase. Finally, a small group of upregulated DEGs that encode putative secreted carboxylic ester hydrolase and hypothetical proteins were present at late time points in angiosperms and not in *P. patens*. Inversely, a group of upregulated DEGs present in *P. patens* at 24 hpi were not differentially expressed in angiosperms, including genes encoding β-glucoside gluco-hydrolase, endo-1,4-beta-xylanase (BcXyn11C), carboxylic ester hydrolase, peroxidase, and an exo-PG, among others.

**Figure 7:**
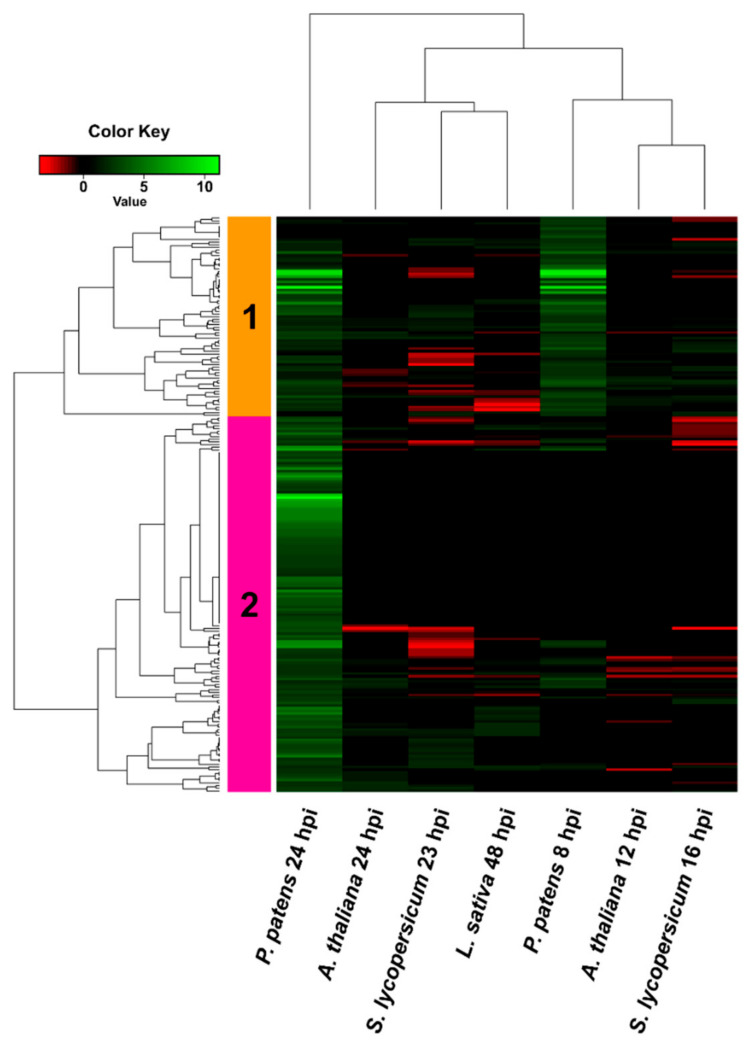
Hierarchical clustering (heat map) showing *B. cinerea* genes only upregulated in *P. patens*. DEGs correspond to the 216 genes that were only upregulated when growing in *P. patens*. Selected DEGs had log2 FC ≥ 2, FDR ≤ 0.05, and were expressed differentially in at least one time point for each plant species. For samples where no significant difference with control was observed (FDR > 0.05) or no data was available, the log2 FC was assigned as cero. See Table S13 for complete information.

We further analyzed in more detail the 216 *B. cinerea* DEGs that were only upregulated in *P. patens* and showed very low or no expression in angiosperms. These DEGs were distributed in two clusters (Figure 7, Table S13): cluster 1 contained upregulated *B. cinerea* DEGs at 8 hpi and 24 hpi, while cluster 2 was mainly composed of upregulated DEGs at 24 hpi.

In cluster 1, four upregulated DEGs showed very high expression levels at 8 and 24 hpi, and encoded a D-malate dehydrogenase, a glyceraldehyde-3-phosphate dehydrogenase (GAPDH), a D-glycerate 3-kinase and a MFS transporter. Interestingly, 22 additional genes encoding putative fungal MSF transporters were upregulated in *P. patens* and in almost all cases expression was not detected or was repressed in angiosperms. Among other upregulated *B. cinerea* genes that were only upregulated in *P. patens* we found carboxylic ester hydrolases, cellobiose dehydrogenase, beta-glucosidases, a peroxidase, a polyketide synthase (BcPks8), and a high number of hypothetical proteins. Additionally, six Zn(2)-C6 transcription factors, including the BcGaaR GalA regulator involved in D-galacturonic acid utilization by *B. cinerea* [47], were upregulated during *P. patens* infection and not in angiosperms. Interestingly, Bcaba2 encoding a Cytochrome P450 monooxygenase, which is an ABA biosynthesis cluster protein, was only upregulated at 24 hpi in *P. patens* tissues.

**Figure 8:**
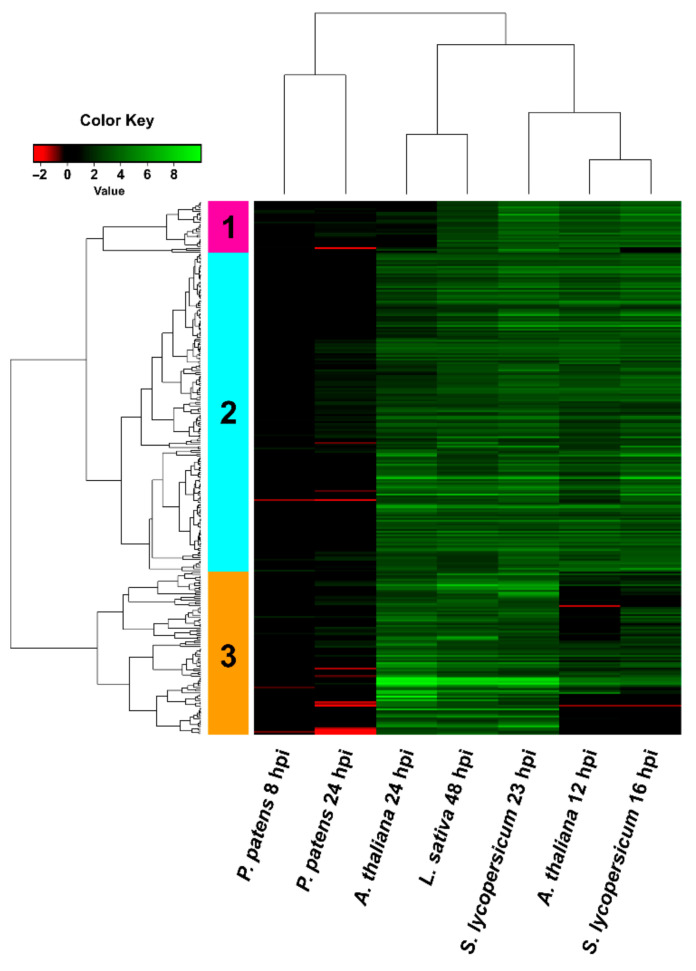
Hierarchical clustering (heat map) showing *B. cinerea* DEGs only upregulated in angiosperms. DEGs correspond to the 288 genes that were upregulated in all angiosperms and not in *P. patens*. Selected DEGs had log2 FC ≥ 2, FDR ≤ 0.05, and were expressed differentially in at least one time point for each plant species. For samples where no significant difference with control was observed (FDR > 0.05) or no data was available, the log2 FC was assigned as cero. See Table S14 for complete information.

Among the 288 *B. cinerea* DEGs that were only upregulated in angiosperms (Figure 4b), we focused on those that were not detected or showed very low expression levels in *P. patens* (Figure 8; Table S14). Interestingly, among them we detected most genes of the botrydial gene cluster at least at one time point, including Bcbot1, Bcbot3, Bcbot4 and Bcbot5. In addition, Bcbot2 was highly upregulated in all angiosperms, while in *P. patens* an induction below log2 FC 2 was observed at 24 hpi. The major positive regulator of botrydial synthesis, the transcription factor Bcbot6 (Bcin12g06420), and a dehydrogenase (Bcbot7; Bcin12g06430), were only upregulated in the three angiosperms. Other fungal upregulated DEGs that were only detected in angiosperms included genes encoding glutaredoxins, GSTs, superoxide dis-mutases (SOD), other oxidoreductase related proteins, carboxyl ester hydrolases, transcription factors including Nmra and bZIP, and hypothetical proteins, among others. Taken together, these results reveal that several *B. cinerea* genes that play important roles in host infection have different expression patterns in the plant species analyzed.

Finally, we looked into *B. cinerea* DEGs that were only downregulated in *P. patens* or in angiosperms (Figure S4, Tables S15 and S16). In total 111 and 93 fungal DEGs were only downregulated in *P. patens* or angiosperms, respectively. From those, only two genes that were downregulated in angiosperms showed increased expression in *P. patens* that was close to Log2 FC=2 (Bcin06g02530: DUF2235 domain-containing protein and Bcin05g05110: hypothetical protein). In contrast, several genes that were downregulated in *P. patens* were upregulated in all angiosperms, including Bcbot4, and two genes encoding a hydrolase EHN domain-containing protein and a PKS. Other DEGs that were downregulated in *P. patens* and showed increased expression in some of the angiosperms included those encoding the aspartic proteinase Bccap1, two ABC transporters, an acyl transferase and several hypothetical proteins. Finally, Bcboa3-5 were downregulated in *P. patens* and showed no expression in most angiosperms. Taken together, our results show that common and differential expression patterns of genes with roles in virulence and infection exist between *P. patens* and angiosperms.

## 4. Discussion

Transcriptional profiling during the *B. cinerea* infection process has been performed in several angiosperms [38,39,40,48,49,50,51]. However, no information related to the molecular mechanisms used by *B. cinerea* during moss invasion is available. Mosses interact with a variety of fungal pathogens in nature and under controlled conditions in the laboratory [52,53]. To get insights into *B. cinerea* infection and virulence strategies developed during moss colonization, we performed a global expression profiling of different infection stages. Expression patterns of upregulated genes and GO enrichment analysis indicated that infection of moss tissues by *B. cinerea* depends on ROS generation and detoxification, transporter activities, plant cell wall degradation and modification, toxin production and probable plant defense evasion by effector proteins. Moreover, a comparison with available RNAseq data during angiosperm infection, including *A. thaliana*, *S. lycopersicum* and *L. sativa*, suggests that *B. cinerea* has probably virulence and infection functions that are used in all hosts, while others are more specific to *P. patens* or angiosperms.

ROS production is important for *B. cinerea* virulence during plant invasion and increased levels of ROS in plant cells are beneficial to the pathogen, leading to accelerated colonization of host tissue [8]. We have previously observed that during *P. patens*-*B. cinerea* interaction, ROS are produced in *P. patens* cells after hyphal contact, probably resembling an oxidative burst response, and also in hyphal tips in contact with moss cells [26]. Similarly, in angiosperms, generation of H_2_O_2_ in the tip of the penetration pegs that breach the cuticle has been described [54]. ROS might assist in host invasion by providing substrates for oxidases that can modify the cuticle [9]. Moreover, endogenous ROS play crucial roles in *B. cinerea* conidial germination and host penetration [55,56]. Here, we show that oxidoreductase activities are enriched in upregulated fungal genes during early *P. patens* infection (4 hpi), including genes encoding BcniaD and BcniiA, that are further upregulated during the entire time course of infection analyzed. Similarly, *BcniaD* and *BcniiA* are both upregulated during *A. thaliana*, *S. lycopersicum* and *L. sativa* infection, but only at late time points. The relevance of this finding needs further investigation, since the production of nitric oxide (NO) by *B. cinerea* probably promotes fungal colonization of the plant tissue, although nitrate reductase is not the main system responsible for the production of NO in this pathogen [57]. Oxidoreductase processes continued during *P. patens* infection at 8 and 24 hpi, and genes encoding laccase, quinone reductase, GMC oxidoreductase, peroxidases, glutathione peroxidase (Bcgpx3), GST, and different types of oxidoreductases were upregulated. Laccase, quinone reductase and GMC oxidoreductase can be a source of ROS production needed for *B. cinerea* infection, while peroxidases, glutathione peroxidases as well as other detoxification enzymes may act to control ROS levels [58]. In addition, Bcin12g02910 encoding a cellobiose dehydrogenase, that is secreted at early time points of infection and could be responsible for ROS generation by *B. cinerea* [59], was upregulated during *P. patens* infection. Similarly, during *B. cinerea* colonization of *A. thaliana*, *S. lycopersicum* and *L. sativa*, a high number of ROS related genes were upregulated, including peroxidases, GSTs, catalases, SODs and different oxidoreductases. Consistently, oxidoreductase activities in the secretome of several *Botrytis* species, including *B. cinerea*, represented 10% of the total secretome, highlighting the importance of these activities during fungal infection [48].

ROS production contribute to host cell death, favoring invasion and further colonization of *B. cinerea* in the dead host tissue [60]. This pathogen facilitates host cell death by producing toxins, including botrydial and botcinic acid [17,18]. We only observed increased expression of fungal genes involved in botcinic acid production, including Bcboa3-7 and Bcboa9, during *P. patens* infection when we compared 24 hpi with 8 hpi, suggesting the involvement of this toxin in moss infection. Interestingly, genes encoding the biosynthesis enzymes of botrydial [61], were upregulated in *A. thaliana*, *S. lycopersicum* and *L. sativa*, while in *P. patens* only Bcbot2 encoding the sesquiterpene cyclase showed a slight induction. Other genes of the botrydial cluster, including Bcbot1, Bcbot3 and Bcbot4 that encode cytochrome P450 proteins, and Bcbot5 encoding a putative acetyl transferase, were not detected or were repressed. In addition, the major positive regulator of botrydial synthesis Bcbot6 that encode a putative Zn(II)2Cys6 transcription factor, and the dehydrogenase Bcbot7 that might be involved in the conversion of botrydial to dihydrobotrydial [62,63], were only upregulated in angiosperms and not in *P. patens*. The fact that botrydial related genes were not upregulated during moss infection was not expected. However, it was previously shown that mutations in botrydial biosynthetic genes and the Bcbot6 regulator did not alter the development and virulence of *B. cinerea*, which is in accordance with a redundant role of botcinic acid and botrydial in virulence of this fungal pathogen [64,65]. Other genes encoding secreted fungal proteins with necrosis-inducing activity such as NEP1 and NEP2 [66], were upregulated in *P. patens* and most analyzed angiosperms. An HR-like response with similar features as in angiosperms has been observed in *B. cinerea*-infected *P. patens* tissues [26], and these proteins may contribute to induce PCD in moss for fungi benefit. In addition, upregulation of several hydrolase-encoding genes during *P. patens* infection, such as Bcpg2, Xyn11A and xyloglucanase BcXYG1, with known cell death inducing activities in angiosperms [43,67,68,69], may contribute to moss cell death and facilitate fungal infection. These three hydrolases are able to induce a necrotic response independent of their catalytic activity, and Bcpg2 and Xyn11A are commonly upregulated genes in all analyzed plant hosts.

As expected for a pathogen with a necrotrophic lifestyle, we show that a large number of *B. cinerea* genes upregulated during *P. patens* and angiosperms infection, encode enzymes with hydrolytic activities. Consistently, hydrolase activity was reported as the most common molecular function of the *B. cinerea* secretome and other *Botrytis* species (approximately 25% of the total secretome) [48]. The production of extracellular proteins is shown to be regulated at the transcriptional level, suggesting a fine-tuning of *B. cinerea* secretome according to conditions that contribute to accomplish successful infection [70]. A high proportion of the upregulated fungal genes in *P. patens* and angiosperms encode CAZymes, including cutinases and PCWDEs with a role in host tissue breakdown and pathogenesis. Many of these enzymes were also detected in different studies of *B. cinerea* secretomes [59,70,71,72,73]. These extracellular enzymes are potential virulence factors that could play important roles in facilitating hyphal growth by softening the host tissues and converting complex plant material available for consumption [74]. Our expression profiles showed that the highest proportion of CAZymes genes were upregulated at late time points of infection, which is consistent with higher maceration rates and cell death of plant tissues, which are needed for effective colonization.

The initial interaction between plants and *B. cinerea* occurs at the plant cuticle, and upregulation of three cutinase-encoding genes in moss tissues suggests that as in other plants [9,15,16,75], these enzymes play a role in breaching *P. patens* cuticle for host penetration. Penetration pores were detected on the surface of *P. patens* cell walls and in intercellular spaces that are colonized by infection hyphae that subsequently invaded leaf cells [27]. Cutinases may be involved in gametophore colonization since fungal penetration pegs penetrate the tissues directly through leaf cell walls [53], and protonema cells have no cuticle [76]. In addition, some *B. cinerea* cutinases show high homology to acetylxylanesterases [77], and could be involved in degradation of xylans in moss tissues. Moreover, the expression pattern of cutinases varied among angiosperms infection and while BccutB was only upregulated in *S. lycopersicum*, other cutinases were expressed in more than one plant. Consistently, previous studies have shown that the expression levels of these enzymes depended on the host tissues [77]. Our results also revealed the presence of common upregulated genes encoding secreted CAZymes in all host plants, as well as CAZymes genes that were detected in some but not all plants hosts. Bcpg2, Bcpg6, two pectin lyases, a pectate lyase, rhamnogalacturonan acetyl-esterase, α-L-rhamnosidase, α-galactosidase, β-galactosidase, α-N-arabino-furanosidase, and Bcxyn11A were upregulated in all plant host. In accordance, PGs, pectin lyases, pectate lyases and Bcxyn11A are essential for virulence and fungal colonization into host tissues [69,75,77,78]. *P. patens* infection showed the highest number of upregulated genes encoding pectin and pectate lyases (8 genes), followed by *S. lycopersicum* infection (6 genes), and finally *A. thaliana* and *L. sativa* infection (4 genes). Moreover, several upregulated DEGs were present in *P. patens* but were not detected in angiosperms, including genes encoding β-glucoside gluco-hydrolase, BcXyn11C, a GPI anchored protein; poly(β-D-mannuronate) lyase, an exo-PG, a glycoside hydrolase and a cellobiose dehydrogenase, among others. In addition, several *B. cinerea* DEGs appeared to be preferentially expressed in one or two specific angiosperms. Remarkably, induced expression of secreted CAZymes could be related to cell wall composition in different plant species [77]. The cell walls of mosses and angiosperms are mainly composed of the same classes of polysaccharides with the exception of xylogalacturonan, which has not been detected in *P. patens*, and some differences exist inside chain composition and structure [79,80]. HG, β-1,4-galactan, α-1,5-arabinan, desterified pectin and RG-I have been identified in *P. patens* and some moss species may contain an RG-II-like polysaccharide [80]. In *P. patens*, the main upregulated *B. cinerea* DEGs were related to pectin homogalacturonan and XyG backbone-degrading enzymes. Furthermore, upregulation of Bclga1, Bclgd1 and Bcgar2 involved in the catabolic pathway of the final product released from pectin degradation, D-galacturonic acid, suggest that pectin depolymerizing and utilization of D-galacturonic acid is important as a carbon nutrient for *B. cinerea*, allowing efficient colonization of *P. patens* tissues. BcGaaR encoding a Zn2Cys6 transcription factor involved in D-galacturonic acid utilization [47], was also upregulated in moss tissues. *B. cinerea* mutants in Bclga1, Bclgd1 and Bcgar2, have affected virulence on *N. benthamiana* and *A. thaliana* leaves, but not on *S. lycopersicum*, indicating host-specific virulence [81]. Interestingly, this difference was correlated with the amount of D-galacturonic acid present in these hosts since *S. lycopersicum* has lower D-galacturonic acid content than *A. thaliana* and *N. benthamiana* leaves. At 24 hpi, upregulated DEGs encoded enzymes involved in HG, XyG backbone, RG-I backbone, xylan backbone, mannans, cellulose, and side-chains/adducts degradation and modification. Upregulation of Bcpme2 in *P. patens* and most angiosperms may facilitate the action of PGs by demethylating pectin to pectate. *B. cinerea* cellulases and hemi-cellulases (xylanases)-encoding genes were also upregulated during *P. patens* and angiosperms colonization. BcAra1 encoding an endo-arabinanase that carries out the breakdown of arabinan [82], was upregulated at late time points during *P. patens*, *A. thaliana* and *S. lycopersicum* infections. Interestingly, BcAra1 plays an important role during infection of *A. thaliana* where mutants provoke smaller lesions, while in *N. benthamiana* lesion size did not differ compared to the wild type strain, indicating that Bcara1 plays a host-dependent role in the virulence of *B. cinerea* [82]. Expression levels of BcAra1 in *N. benthamiana* were much lower than in *A. thaliana* indicating regulation at the transcriptional level that serves as a determinant of disease progression. Taken together, our results are consistent with host-dependent expression of genes related to cell-wall degradation and modification by *B. cinerea*.

Two of the highest expressed *B. cinerea* genes during *P. patens* infection encoded a malate dehydrogenase and GAPDH. Interestingly, malate dehydrogenase catalyzes the reversible conversion of oxalacetate and malate, and oxalacetate is a precursor of the pathogenicity factor oxalic acid [83]. Oxalic acid facilitates *B. cinerea* infection through different mechanisms, including enhancement of PGs activity to promote cell wall degradation, suppression of the plant oxidative burst, induction of HR, inhibition of plant–protection enzymes, and alteration of the cellular redox status in the plant [12]. Consistently, malate dehydrogenase has been identified in the secretome of different *B. cinerea* strains [84]. GAPDH functions in the glycolytic cycle and it may serves as a virulence factor as has been proposed for several pathogenic fungi [85]. In addition, other virulence genes were upregulated during *P. patens* infection, including BcCFEM1, which has a putative GPI modification site and is required for virulence and tolerance to osmotic and cell wall stress [43]. Other genes encoding CFEM domain-containing proteins were upregulated in the different hosts, which is consistent with their potential role in pathogenicity [86,87]. Upregulated genes encoding secreted proteins with putative virulence functions during *P. patens* and angiosperm infection included different CND proteins. The calcium/calcineurin signaling pathway controls the botrydial gene cluster, and calcineurin phosphatase has an essential role in virulence and was shown to be involved in *B. cinerea* appressorium formation [10,88]. Secreted proteinases and peptidases have been reported as virulence factors involved in host-tissue invasion and pathogenicity, and different types of fungal proteases were upregulated in *P. patens* and angiosperms, including aspartic proteinases [89]. Consistently, a high number of proteases were found in different *B. cinerea* secretomes [42,48,59]. Several studies have demonstrated the role of secreted effector genes in the establishment of *B. cinerea* infection via suppression of plant defense [42]. We identified several candidate effectors genes that were upregulated during *P. patens* infection and could interfere with moss defenses. They include known effectors such as BcNEP1 and BcSSP2 [90], two ribonucleases and three hypothetical proteins, including Bcin04g03920, which is highly expressed in *P. patens*, *A. thaliana* and *S. lycopersicum*. Ribonucleases are known effectors in other necrotrophic pathogens, such as *Blumeria graminis*, where they bind to host ribosomes and inhibit the action of plant ribosome-inactivating proteins that are known components of plant immune responses that lead to host cell death [91].

Plants perceive pathogens by detecting pathogen-associated molecular patterns (PAMPs) through pattern recognition receptors [92]. Several secreted enzymes of *B. cinerea* are recognized by plant leucine-rich repeat receptors, including PGs [93], and BcXYG1 [42], leading to activation of plant defense. Interestingly, *P. patens* induces rapidly the expression levels of a high number of genes encoding these type of receptors during *B. cinerea* infection [28]. However, further research is needed to understand if some of these receptors recognize PGs and other fungal PAMPs in moss tissues. Moreover, we observed that two genes encoding putative plant pectin methyl-esterase inhibitor were upregulated in *P. patens* after *B. cinerea* colonization [28]. These inhibitors could be involved in making the wall more resistant to degradation by fungal enzymes by increasing methylated pectin in the cell wall [94]. In response to plant defense activation, *B. cinerea* has developed different strategies to interfere with plant immunity, including secretion of hydrolases to detoxify antifungal secondary metabolites produced by the host. *B. cinerea* is capable of degrading stilbene phytoalexins [95] from grapevines, α- tomatine [96] from *S. lycopersicum*, and metabolize scopoletin from tobacco [97]. Moreover, cruciferous phytoalexins were detoxified by *B. cinerea* via either oxidative degradation or hydrolysis [98]. In addition, upregulation of genes encoding peptidases and proteases during *P. patens* and angiosperms infection, leads to amino acids release for fungal growth, degradation of plant cell wall proteins and defense proteins [59]. Plant pathogens, have transporters, such as ATP-binding cassette (ABC) and MFS transporters, involved in the excretion of plant compounds that are toxic for the pathogen and are produced by the host as a defensive strategy [99]. These transporters contribute to virulence and are especially useful for pathogens with a broad host range [99], including *B. cinerea*. Here, we show that during *P. patens* infection, *B. cinerea* induces the expression of a high number of genes encoding putative MFS transporters, some of which are very highly induced (Bcin07g06720 and Bcin09g05570). Interestingly, most of these MSF-transporter encoding genes were only upregulated during *P. patens* infection and not in angiosperms. The large number of MSF transporters gene members in the *B. cinerea* genome, 286 and 282 genes in strain T4 and strain B05.10, respectively, highlight their importance in counteracting the effect of antimicrobial compounds [100]. Consistently, a MFS and an ABC transporter contribute to virulence in *B. cinerea* and increase tolerance to glucosinolates and camalexin respectively, which are produced by *A. thaliana* as defensive compounds [100,101]. Moreover, Bcmfs1 is involved in tolerance to antifungal compounds such as camptothecin and cercosporin [102]. Our results suggest that MFS transporters could play an important role in *P. patens*–*B. cinerea* interaction, although further studies are needed to reveal whether these transporters are involved in detoxification of moss specific metabolites. Until now, only few compounds with antifungal activity have been identified in *P. patens* [103], and due to their requirement in improved defenses to biotic challengers during plant colonization, these metabolites are of particular interest.

## 5. Conclusions

Our transcriptional profiling of *B. cinerea*-infected plants demonstrated that common molecular mechanisms were involved in fungal virulence and the infection process in *P. patens* and angiosperms. However, differences in expression patterns of fungal CAZymes genes and putative MFS transporter genes between hosts revealed that some infection functions are specific to *P. patens* or angiosperms infections. A deeper understanding of the plant cell wall targets, as well as the identity of metabolites with antifungal activities of the encoded proteins by *P. patens*, will certainly increase our knowledge of bryophyte-fungal interactions. In addition, the use of *B. cinerea* mutants will contribute to uncover the molecular pathogenic mechanisms and regulatory network used by this pathogen during moss infection and their involvement during coevolution of pathogens with land plants.

## Supplementary Materials

The following are available online at https://www.mdpi.com/2309-608X/7/1/11/s1, Figure S1: *B. cinerea* infection of *P. patens* tissues. Infection progress during time is shown; (a) spore germination at 4 hpi, (b) germ tubes elongation at 8 hpi, (c) proliferation of mycelium at 24 hpi, (d) appresorium formation at 8 hpi, (e) hyphae colonizing a leaf cell, and (f) hyphae colonizing a protonemal cell. Symptom development at 8 hpi (h), 24 hpi (i) and 24 h water-treated moss colonies (g) are shown. The arrow in (d) indicate an appresorium. The scale bar in a-f represents 10 μm and 0.5 cm in g-i. Figure S2: Enriched gene ontology (GO) terms of *B. cinerea* DEGs. The top 15 enrichment GO terms were chosen according to their statistical significance (−log10 FDR). GO enrichment are shown for upregulated and downregulated *B. cinerea* DEGs at 4, 8 and 24 hpi. See Table S5 for complete information. Figure S3: Analysis of *B. cinerea* RNA-Seq data during *P. patens*, *A. thaliana*, *S. lycopersicum* and *L. sativa* infection by principal component analysis (PCA). Colored dots denote each biological replicate. Figure S4: *B. cinerea* downregulated DEGs during *P. patens* and angiosperm infection. (a) Venn diagram of *B. cinerea* downregulated DEGs in different plant species, showing overlap of fungal repressed genes. *P. patens*: sum of *B. cinerea* DEGs at 8 and 24 hpi; *A. thaliana*: sum of *B. cinerea* DEGs at 12 and 24 hpi; *S. lycopersicum*: sum of *B. cinerea* DEGs at 16 and 23 hpi; and *L. sativa*: *B. cinerea* DEGs at 48 hpi. For Venn diagram, DEGs had log2 FC ≤ −2, FDR ≤ 0.05, and were expressed differentially in at least one time point for each plant species condition. (b) Hierarchical clustering (heat map) showing *B. cinerea* genes only downregulated in *P. patens*. Fungal DEGs corresponds to the 111 genes that were only downregulated in *P. patens*. (c) Hierarchical clustering showing *B. cinerea* genes only downregulated in angiosperms. Fungal DEGs corresponds to the 93 genes that were only downregulated in angiosperms. Table S1: List of qPCR primers used in this study. Table S2: Summary of mapped reads of *B. cinerea* in the RNA-Seq libraries. 1–3 indicate the three biological replicates of different plants species at the indicated time points. Table S3: List of *B. cinerea* differentially expressed genes (DEGs) during *P. patens* infection at 4, 8 and 24 hpi. Table S4: Enriched gene ontology (GO) terms (over-representation) for biological processes (BP), molecular function (MF) and cell compartment (CC) at 4, 8 and 24 hpi. Table S5: Validation of RNA-Seq data by qPCR assay: correlation of log2 FC values for 16 genes obtained by RNA-Seq and qPCR. Gene name and identification, log2 fold change from RNA-seq and RT-qPCR analysis and description are provided. Table S6: List of *B. cinerea* upregulated DEGs encoding secreted proteins during infection of *P. patens* at 4, 8 and 24 hpi. Table S7: Summary of numbers of *B. cinerea* analyzed genes. Table S8: List of *B. cinerea* differentially expressed genes (DEGs) during *A. thaliana* infection at 12 and 24 hpi. Table S9: List of *B. cinerea* differentially expressed genes (DEGs) during *S. lycopersicum* infection at 16 and 23 hpi. Table S10: List of *B. cinerea* differentially expressed genes (DEGs) during *L. sativa* infection at 48 hpi. Table S11: List of common upregulated genes in all plants species. *B. cinerea* DEGs were included when log2 FC was ≥ 2 and FDR ≤ 0.05 in at least one time point of each plant species. For samples where no significant difference with control was observed (FDR > 0.05) or no data was available, the log2 FC was assigned as cero. Table S12: List of genes encoding putative secreted *B. cinerea* proteins in *P. patens*, *A. thaliana, L. lycopersicum* and *L. sativa* at the indicated time points of infection. DEGs were considered when FDR was ≤0.05. For samples where no significant difference with control was observed (FDR > 0.05) or no data was available, the log2 FC was assigned as cero. Table S13: List of 96 *B. cinerea* DEGs that were only upregulated in *P. patens*. DEGs were considered when log2 FC was ≥ 2 and FDR ≤ 0.05 in at least one time point of each plant species. For samples where no significant difference with control was observed (FDR > 0.05) or no data was available, the log2 FC was assigned as cero. Table S14: List of 288 *B. cinerea* DEGs that were only upregulated in all angiosperms and not in *P. patens*. DEGs were considered when log2 FC was ≥ 2 and FDR ≤ 0.05 in at least one time point of each plant species. For samples where no significant difference with control was observed (FDR > 0.05) or no data was available, the log2 FC was assigned as cero. Table S15: List of 111 *B. cinerea* DEGs that were only downregulated in *P. patens*. DEGs were considered when log2 FC was ≤ −2 and FDR ≤ 0.05 in at least one time point of *P. patens*. For samples where no significant difference with control was observed (FDR > 0.05) or no data was available, the log2 FC was assigned as cero. Table S16: List of 93 *B. cinerea* DEGs that were only downregulated in all angiosperms and not in *P. patens*. DEGs were considered when log2 FC was ≤ −2 and FDR ≤ 0.05 in at least one time point of each plant species. For samples where no significant difference with control was observed (FDR > 0.05) or no data was available, the log2 FC was assigned as cero.
